# Exploration of New Lipid Nutrients and Their Characterization in Herbal Teas Using Non-Targeted Liquid Chromatography–Mass Spectrometry

**DOI:** 10.3390/foods13121877

**Published:** 2024-06-14

**Authors:** Md Abdul Malek, Siddabasave Gowda B. Gowda, Rachana M. Gangadhara, Divyavani Gowda, Shu-Ping Hui

**Affiliations:** 1 Graduate School of Global Food Resources, Hokkaido University, Kita-9, Nishi-9, Kita-ku, Sapporo 060-0809, Japan; mdabdul.malek.v0@elms.hokudai.ac.jp (M.A.M.); rachanachem03@gmail.com (R.M.G.); 2 Faculty of Health Sciences, Hokkaido University, Kita-12, Nishi-5, Kita-ku, Sapporo 060-0812, Japan; divyavani@hs.hokudai.ac.jp

**Keywords:** liquid chromatography, mass spectrometry, herbal tea, lipids, FAHFAs, correlation analysis

## Abstract

Herbal teas are blends of leaves, seeds, fruits, and flowers from various plants that provide relaxation, anti-inflammatory benefits, and immune system support for conditions such as diabetes and asthma. Despite their health benefits, comprehensive lipidomic data on herbal teas are limited in the literature. We used non-targeted liquid chromatography–linear ion trap orbitrap mass spectrometry to identify and correlate the lipid species in the following six herbal tea samples: fennel, ginger, juniper, lemon peel, orange peel, and rosehip. A total of 204 lipid molecular species were identified, and multivariate analysis revealed a significant difference between lipid species in herbal teas. Saturated fatty acids (SFAs) and polyunsaturated fatty acids (PUFAs) are significantly abundant in juniper, including ω-3 and ω-6 fatty acids, followed by fennel. Cluster correlations showed that ginger contained mainly sphingolipids and lysophospholipids, whereas fennel was rich in phospholipids. No significant variations in the content of triacylglycerols were observed in any of the herbal teas analyzed. The ratio of PUFAs to SFAs in herbal teas showed that orange peel had the highest ratio, followed by lemon peel and fennel, indicating their potential health benefits. In addition, using high-resolution mass spectrometry, various lipids such as fatty acid esters of hydroxy fatty acids and N-acyl-lysophosphatidylethanolamines were identified and characterized in these herbal teas. This study provides a comprehensive lipid analysis and detailed characterization of lipids in six herbal teas, highlighting their plausible applications in the field of nutrition and various food industries for the development of functional foods.

## 1. Introduction

Herbs have been widely used in the form of herbal teas for a long time because of their potential health benefits. Herbal tea consumption has multiple benefits for human health, including anti-inflammatory effects and boosting the immune system against disease [1]. Fennel is an economically important aromatic plant that is a good source of nutrition and is used as a spice in cooking and as herbal medicine [2]. Fennel seeds have been reported to have antispasmodic, antiseptic, carminative, and antiulcer properties, and are used to treat various diseases such as gastrointestinal disorders, kidney stones, diarrhea, vomiting, and neurological disorders [3]. Previous studies have shown that fennel contains 21 fatty acids, including linoleic and alpha-linolenic acids, and high levels of ω-6 and ω-3 fatty acids subsisted in fennel using high-performance liquid chromatography and gas chromatography with flame ionization detection analysis [4]. Ginger, a flowering plant, was previously reported to have antioxidant and hepatoprotective activities against alcoholic fatty liver disease [5]. Its extracts have widely been used in Chinese medicine for a long time, with physiological effects on the human body, such as reducing nausea [5,6], increasing intestinal and gastric function [7], inhibiting cardiovascular disease [8], improving liver function [9], and inhibiting cancer cell proliferation [10].

Among the various applications, ginger is used in the food, spice, cosmetic, and beverage industries. Citral, a major component of its extract, has been shown to have antioxidant, anticancer, anti-inflammatory, anticandidal [11,12], and inhibition effects on leukotriene biosynthesis by blocking 5-lipoxygenase [13]. Previously, the lipid metabolites detected in ginger were lysophosphatidylcholines (LPCs), including LPC 16:0, LPC 18:1, LPC 18:2, and LPC 18:3, as determined using gas chromatography–mass spectrometry (GC–MS), and targeted/non-targeted analysis using liquid chromatography–mass spectrometry (LC–MS) [14]. Juniper, an evergreen shrub, grows in forests, especially in Western and Eastern Siberia and in Europe, and has a wide range of biological activities and industrial applications [15]. It is recognized as a traditional medicine with diuretic, anti-inflammatory, antifungal, analgesic, hepatoprotective, antibacterial, and antioxidant properties [16]. Some long-chain saturated fatty acids ranging from nonanoic acid (C9:0) to behenic acid (C22:0), unsaturated fatty acids, and hydroxy and dihydroxy fatty acids have been previously reported in juniper extracts, as determined using mass spectrometry [17].

Lemon peel, a by-product of *Citrus limon*, is very important due to its high biotechnological potential and biologically active components such as flavanones including hesperidin and naringin [18,19]. It has various biological properties, such as antimicrobial, antioxidant, anti-stress, and anti-inflammatory properties in humans and ruminants [20]. Its extracts as food derivatives contain fatty acids, especially enhanced conjugated linoleic acids, monounsaturated fatty acids (MUFAs), and polyunsaturated fatty acids (PUFAs) in milk and its derivatives [21,22]. Lipidomic analysis using LC–MS showed that glycerophospholipids (GPs) and sphingolipids (SPs) were the predominant polar lipids in milk, and phosphatidylcholines (PCs) and phosphatidylethanolamines (PEs) were significantly increased in milk by feeding lemon peel extracts to cows [23]. Orange peel is a by-product of the fruit of *Citrus aurantium*, a member of the Rutaceae family, and has biological importance and a variety of industrial uses, including for food, cosmetics, and nutraceuticals [24].

Previous studies have shown that orange peel extracts have antioxidant, anti-inflammatory, and antimicrobial activities of flavonoids [25,26], as well as anti-atherosclerosis and anti-cancer activities [27]. Fatty acids extracted from orange peel include palmitic acid, stearic acid, oleic acid, linoleic acid, linolenic acid, and lignoceric acids, and essential oils, which have an antibacterial activity [28] and insecticidal activity [29]. Rosehip, also known as rose haw, is a fruit of various rose plants used in pharmaceutical and cosmetic industries and has been encapsulated in phospholipid liposomes using the single-step pro-liposome method [30]. Rosehip is also considered for its preventive and curative properties against a wide range of renal, inflammatory, gut, and gastric diseases [31], as well as for its antioxidant, antibacterial, analgesic, mild laxative, diuretic, anti-cancer, and positive effects on dermatoses, ulcers, and skin diseases [32,33]. Various studies have been conducted to identify the chemical composition of components such as polyphenols, flavonoids, fatty acids, and specific lipid classes in ginger, lemon peel, and orange peel samples using GC–MS, LC–MS, and other methods [17,24,25].

Despite the potential health benefits and implications of these herbal teas at an industrial level, descriptive lipidomic data are lacking in previous studies. Most of the studies performed are using the targeted techniques where the analysis was limited to a specific class of lipids, such as fatty acyls. On the other hand, non-targeted analysis has advantages over targeted analysis, particularly in performing a comprehensive analysis of multiple lipid classes at the molecular level and in uncovering novel lipid metabolites. Therefore, we aimed to analyze the total lipid composition of six herbal teas at the molecular species level using a non-targeted LC–MS method. During our analysis, novel lipids were identified and characterized for the first time in these herbal samples.

## 2. Materials and Methods

### 2.1. Sample Information

The six herbal teas, namely Fennel (F)—*Foeniculum vulgare Mill.*, Ginger (G)—*Zingiber officinale*, juniper (J)—*Juniperus communis*, Lemon peel (LP)—*Citrus limon,* Orange peel (OP)—*Citrus aurantum*, and Rosehip (RH)—*Rosa canina*, were purchased from an online shop (https://onlineshop.treeoflife.co.jp, accessed on 4 April 2023). All samples were dried, packaged, and ground to powder using a mortar before the analysis.

### 2.2. Materials

LC–MS-grade reagents such as methanol, isopropanol, chloroform, and ammonium acetate solution (1M) were purchased from Wako Pure Chemicals Industries, Ltd. (Osaka, Japan). The internal standards, oleic acid-d9 and EquiSPLASH LIPIDOMIX, were purchased from Avanti Polar Lipids (Alabaster, AL, USA). The international standard mixture was prepared with a final concentration of 10 µg/mL oleic acid-d9 and 1 µg/mL EquiSPLASH in methanol.

### 2.3. Extraction of Total Lipids from Herbal Teas

The total lipids were extracted from herbal teas using the Bligh and Dyer method, with minor modifications, as has been established previously in our laboratory [34,35]. In brief, 100 mg of the powdered herbal teas were weighed into a 2 mL Eppendorf tube (*n* = 2 for each), which is preloaded with 5–6 zirconium ceramic beads (1.4 mm, catalog no. 15-340-159, Fisherbrand, Madawaska, ME, USA). Then, 1 mL of methanol was added and homogenized for 30 s (×3 cycles) to obtain the uniform mixture. Then, the homogenate was centrifuged at 15,000 rpm for 10 min at 4 °C and the centrifugate was collected into a new vial. For further extraction, 100 µL of homogenate (*n* = 5 each) was placed in a 1.5 mL Eppendorf tube, and then 100 µL of the internal standard mixture, 100 µL of chloroform, and 20 µL of Milli-Q were added and the solution was vortexed for 5 min at 3500 rpm. The single-phase extracts were incubated at room temperature for 2 h and were then concentrated in a centrifugal evaporator at 4 °C overnight. After evaporation, the residue was re-dissolved in 100 µL of methanol with gentle vortexing and was then centrifuged for 10 min at 15,000 rpm at 4 °C. Finally, the lipid extracts were transferred to an LC–MS vial and 20 µL of each sample was injected into the LC–MS equipment to obtain lipid composition data.

### 2.4. Non-Targeted Lipidomic Analysis Using an HPLC–LTQ Orbitrap MS

Total lipidomic analysis was performed using a High-Performance Liquid Chromatography (HPLC) system (Shimadzu Corporation, Kyoto, Japan) that was connected to a linear ion trap-Orbitrap mass spectrometer (Thermo Fisher Scientific Inc., San Jose, CA, USA). The analysis was performed using a reverse-phase Atlantis T3 C18 column (2.1 × 150 mm, 3 µm; Waters, Milford, MA, USA), at an oven temperature of 40 °C and a flow rate of 0.2 mL/min of mobile phase through the column. The mobile phase for LC–MS analysis consisted of A–10 mM aqueous ammonium acetate, B–isopropanol, and C–methanol. A linear gradient flow rate was maintained as follows: 0–1 min 30% of B, 35% of C; 1–14 min 80% of B, 10% of C; 14–27 min 85% of B, 10% of C; and 3 min for column equilibration to the initial condition. Lipidomic analysis using mass spectrometry (MS) was carried out in both negative and positive ionization modes with data-dependent acquisition conditions and the maintenance of identical parameters that were previously reported in our laboratory [36,37]. The MS conditions for the negative ionization mode were as follows: negative electrospray ion source voltage was 3.0 kV; nitrogen sheath gas flow rate was 50 arbitrary units; nitrogen auxiliary gas flow rate was 5 arbitrary units; and full-scan MS was set at *m*/*z* 160–1900 in the Fourier-transform mode with the resolving power of 60,000. High-resolution MS^1^ and MS^2^ mass spectra were obtained using collision-induced dissociation at a collision energy of 40 eV. The MS parameters for the positive mode of ionization were identical to those reported in previous studies [36,37]. The raw data acquired using MS were processed using MS DIAL (version 4.9) and Xcalibur 2.2 software, for the annotation, alignment, peak detection, and identification of the different lipid molecular species. The semi-quantitative results were calculated based on the amount of internal standard added and normalized according to the weight of the sample used for the analysis.

### 2.5. Statistical Analysis

Data were plotted as mean ± standard deviation using Microsoft Excel 2016 and GraphPad Prism version 8 (San Diego, CA, USA). MetaboAnalyst (version 6.0) (https://www.metaboanalyst.ca/, accessed on 6 January 2024) and ClustVis tools (https://biit.cs.ut.ee/clustvis/, accessed on 6 January 2024) were used for data visualizations. 

## 3. Results and Discussion

### 3.1. Total Lipid Extraction in Six Herbal Teas and Their Lipidomic Profiles

**Figure 1:**
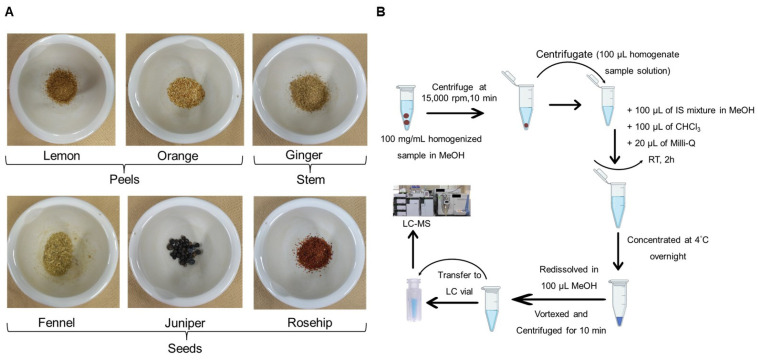
(**A**) Images of the six herbal tea samples used in this study. (**B**) Workflow of total lipid extraction from herbal teas for LC–MS analysis.

The six different herbal teas (fennel, ginger, juniper, lemon peel, orange peel, and rosehip) used for the analysis, as well as their extracted portion, are shown in Figure 1A. The workflow for the total lipid extraction from herbal teas is shown in Figure 1B. Six herbal teas were analyzed using a non-targeted LC–MS technique. The lipidomic analysis of these herbal teas revealed more than 204 lipid molecular species from five major lipid classes including fatty acyls (FAs), glycerophospholipids (GPs), glycerolipids (GLs), sphingolipids (SPs), and sterol lipids (STs). The different lipids were identified according to the LIPID MAPS guidelines, and their identifications were confirmed based on their exact molecular masses and MS/MS spectral behavior with the reference spectra of the MS DIAL software (ver. 4.9). MS DIAL is a freely available software which can perform the annotation, alignment, peak detection, and identification based on the following set parameters: a minimum peak height of 1000 amplitude, a mass slice width 0.1 Da, and a signal intensity 5-fold greater than that of the blank. Each lipid metabolite peak was manually confirmed, and its MS/MS spectral matching was also verified with the inbuilt library spectra. After that, the lipid metabolites list was exported; further, each metabolite peak was manually integrated to obtain the peak area for relative quantification using Xcalibur 2.2 software. The detailed list of lipid species and their amounts in each herbal tea are provided in the Supporting Information Table S1. Since all samples are a great source of bioactive compounds and have a positive impact on human health [2,9,15,20,26], this is the first study on the comprehensive lipid analysis of herbal teas.

### 3.2. Principal Component Analysis (PCA) of Six Types of Herbal Teas

**Figure 2:**
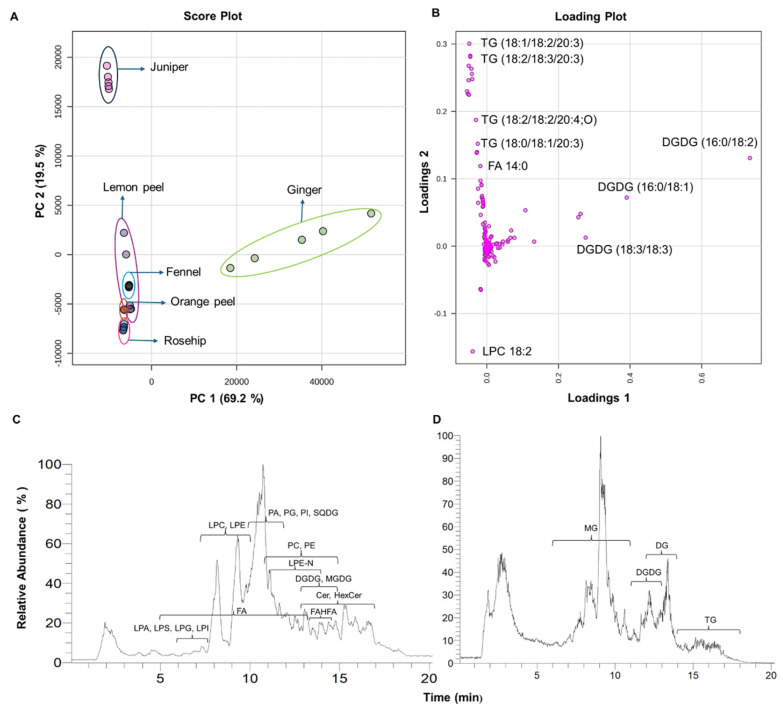
(**A**) Multivariate analysis of lipid composition in six herbal teas. (**A**) Principal component analysis (PCA) score plot of 204 identified lipid molecular species (Tukey’s HSD test with *p* < 0.05). (**B**) PCA loading plot of total lipid metabolites. (**C**) Total ion chromatogram (TIC) acquired in negative ionization mode. (**D**) Total ion chromatogram acquired in positive ionization mode.

The identification and relative quantification of the different lipids were performed using internal standards, and the results were subjected to multivariate analysis, such as Principal Component Analysis (PCA), to demonstrate the distinct differences in lipid composition between the sample groups. The PCA score plot and loading plot of the total lipid species identified from the six herbal teas are shown in Figure 2A,B. The PCA plot showed the clear group separation among the six herbal teas, indicating significant differences in the lipid compositions, with PC2 accounting for 19.5% and PC1 accounting for 69.2% of the variance followed by the loading plot. The plot shows a significant positive deviation for juniper, indicating that juniper has a distinct lipid composition compared to other herbal teas. The loading plot shows the components responsible for the notable separation between groups. The total ion chromatograms (TICs) of herbal teas in negative and positive ionization modes are shown in Figure 2C and Figure 2D, respectively. The lipids such as fatty acyls (FAs); fatty acid esters of hydroxy fatty acids (FAHFAs); phospholipids including phosphatidic acid (PA), phosphatidylcholine (PC), phosphatidylethanolamine (PE), and phosphatidylglycerol (PG); lysophospholipids including lysophosphatidic acid (LPA), lysophosphatidylcholine (LPC), lysophosphatidylethanolamine (LPE), lysophosphatidylglycerol (LPG), lysophosphatidylinositol (LPI), lysophosphatidylserine (LPS), and N-acyl-lysophosphatidylethanolamines (LPE-Ns); sphingolipids such as ceramides (Cers) and hexosylceramides (HexCers); sterols such as stigmasterol hexosides (SG: Hexs); glycerolipids such as digalactosyldiacylglycerol (DGDG), sulfoquinovosyldiacylglyecrol (SQDG), and monogalactosyldiacylglycerol (MGDG); monoacylglycerols (MGs); diacylglycerols (DGs); and triacylglycerols (TGs) were detected in herbal teas.

### 3.3. Distribution of Lipid Compositions in Six Types of Herbal Teas

**Figure 3:**
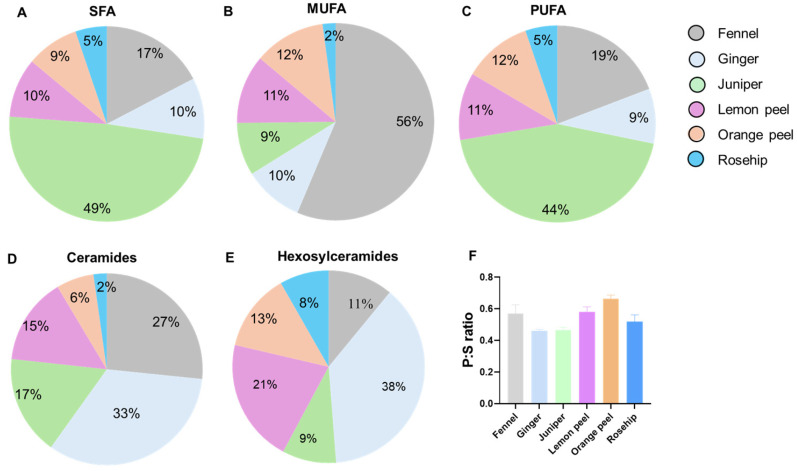
Percentages of the relative concentration of (**A**) saturated fatty acids (SFAs), (**B**) monounsaturated fatty acids (MUFAs), (**C**) polyunsaturated fatty acids (PUFAs), (**D**) Ceramides, (**E**) Hexosylceramides and (**F**) PUFA to SFA (P:S) ratio of six herbal teas.

The percentages of the relative concentrations of free fatty acids (FFAs), SPs, and the ratio of PUFAs to SFAs (P:S) in six herbal teas are shown in Figure 3. The composition of SFAs differed among the herbal teas, with juniper having the highest concentration at 49%, followed by fennel at 17%, and rosehip at the lowest level of 5% (Figure 3A). Figure 3B shows that fennel has the highest amount of MUFAs at 56%, followed by orange peel at 12%, lemon peel at 11%, ginger at 10%, juniper at 9%, and rosehip at 2%. The highest amount of bioactive PUFAs was found in juniper with 44%, followed by fennel with 19%, while ginger, lemon peel, orange peel, and rosehip had 9%, 11%, 12%, and 5%, respectively, as shown in Figure 3C. The relative percentages of Cers and HexCers in herbal teas are shown in Figure 3D and Figure 3E, respectively. Ginger and fennel had the highest percentages of Cers followed by juniper and lemon peel, while rosehip had the lowest value of 2%. On the other hand, ginger and lemon peel had the highest amount of HexCers, with 38% and 21%, consequently followed by orange peel with 13%, while rosehip had the lowest amount with 8%, as shown in Figure 3E. The PUFAs to SFAs P:S ratio is shown in Figure 3F. A high P:S ratio is considered beneficial for cardiovascular health [38]. Among the six herbal teas, orange peel had the highest P:S ratio followed by the lemon peel.

### 3.4. Hierarchical Cluster Correlation Analysis of Lipids Characterized in Six Herbal Teas

**Figure 4:**
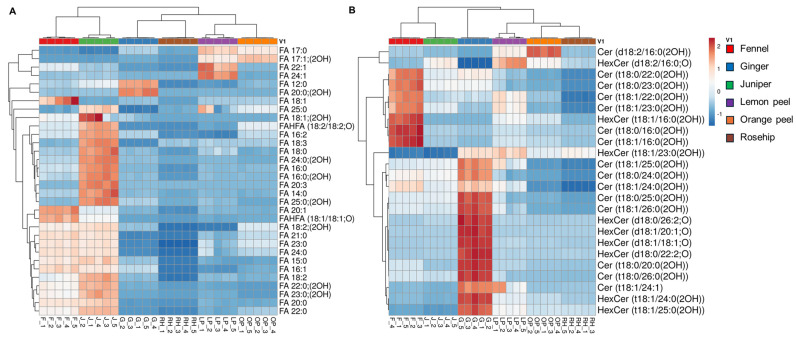
Hierarchical cluster correlation analysis of lipid molecular species, (**A**) free fatty acids and (**B**) Sphingolipids, obtained in this analysis.

The correlation analysis of lipid species identified in six herbal teas was grouped based on the lipid nomenclature, and their relative concentrations are represented in heatmaps. The intense red color indicates higher lipid concentrations, while, conversely, the intense blue color indicates lower lipid concentrations. The cluster correlation analysis of the FFAs is shown in Figure 4A. The results show that the odd-chain fatty acids such as FA 17:0 and FA 17:1; (2OH) are abundant in both lemon peel and orange peel, whereas FA 12:0 and FA 20:0; (2OH) are relatively higher in the ginger sample. The levels of many long-chain SFAs (C > 20) were relatively higher in fennel and juniper. MUFAs such as FA 22:1 and FA 24:1 are higher in lemon peel, whereas FA 18:1, FA 20:1, FAHFA (18:1/18:1; O), and FA 16:1 are relatively higher in fennel compared to other species. Interestingly, PUFAs and their derivatives, including FA 16:2, FA 18:2 (ω-6 fatty acid), FA 18:3 (ω-3 fatty acid), FA 20:3, FA 18:2 (2OH), and FAHFA (18:2/18:2; O), are abundant in the juniper seeds. These results are consistent with a previous study, which showed the occurrence of large amount of PUFAs in juniper seeds [39]. Further, the fatty acids obtained from these herbal teas demonstrated a positive effect on human health when they were consumed as dietary supplements [22,40]. 

The heatmap in Figure 4B shows the relative concentrations of SPs in the herbal teas, with ginger and fennel standing out in red, indicating higher concentrations, whereas orange peel and rosehip are in blue, indicating lower concentrations. Ceramides such as Cer (d18:2/16:0 (2OH)) are abundant in orange peel, whereas HexCer (d18:2/16:0; O) is more abundant in lemon peel compared to orange peel and juniper. Among most of the Cers and HexCers characterized, both are highly abundant in ginger followed by the fennel samples. Several studies have demonstrated that ginger and its extracts possess extensive pharmacological activities, including antioxidant, anti-inflammatory, antitumor, antibacterial, and anti-hepatotoxicity properties due to their distinct lipid species and chemical compositions [41]. Also, plant-derived ceramides are believed to have a positive impact on improving the skin’s epidermal barrier function [42].

**Figure 5:**
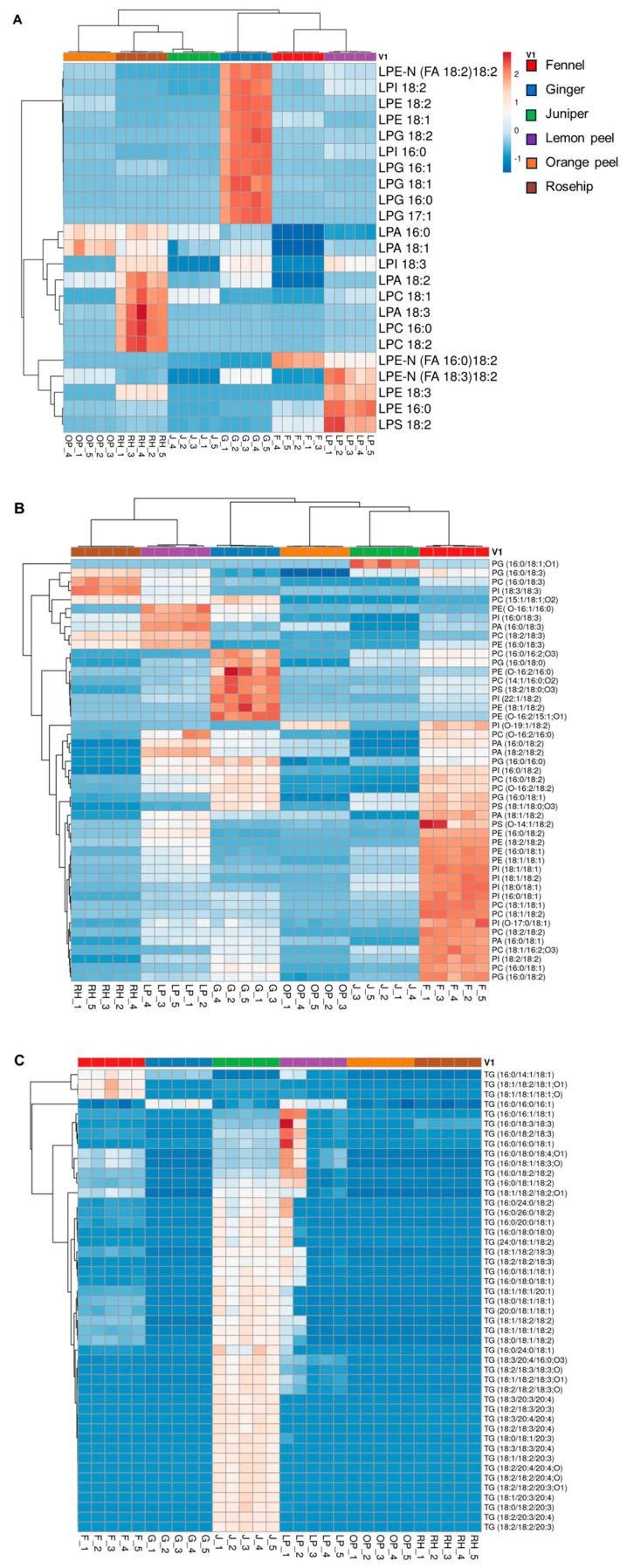

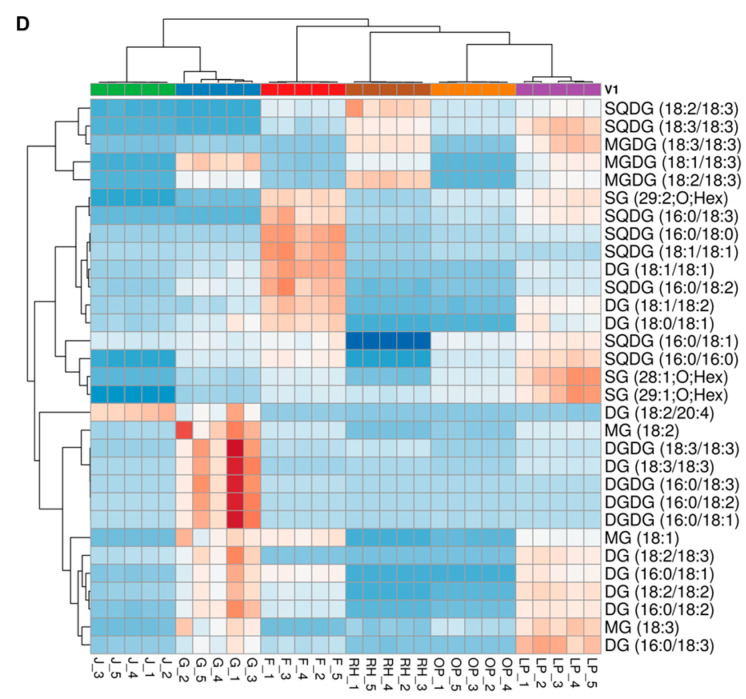
Hierarchical cluster correlation analysis of glycerophospholipids (GPs): (**A**) lysophospholipids; (**B**) phospholipids, glycerolipids (GLs), and sterol lipids (ST); (**C**) triacylglycerols; and (**D**) MGs, DGs, DGDGs, SGs (sterol lipids), SQDGs, and MGDGs.

In addition, hierarchical cluster analysis of GPs including lysophospholipids and phospholipids, as well as glycerolipids and sterol lipids, are visualized in the heatmap in Figure 5A, Figure 5B, Figure 5C, and Figure 5D, respectively. Lysophospholipids were highest in ginger followed by rosehip and lemon peel, while most of the phospholipids were highest in fennel followed by ginger and lemon peel (Figure 5A,B). Lysophospholipids such as LPI 18:2, LPI 16:0, LPE 18:2, LPE 18:1, LPG 18:2, LPG 16:1, LPG 18:1, LPG 16:0, and LPG 17:1 are abundant in ginger. Rosehip contains higher levels of LPA 18:3, LPC 16:0, LPC 18:1, and LPC 18:2, while lemon peel has higher levels of LPE 16:0 and LPS 18:2, respectively. Fennel has significantly lower levels of LPA 16:0, LPA 18:1, and LPA 18:2. Phospholipids such as PC, PG, PE, PI, PA, and PS are found in higher concentrations in fennel, followed by ginger and lemon peel. In most cases, orange peel and juniper showed lower concentrations, as is shown in Figure 5B. TGs were found in lower concentrations except in juniper, which showed a slight red color, and very few lipids showed an intense red color in the lemon peel sample with higher concentration, as shown in Figure 5C. The hypoglycemic and hypolipidemic effects of juniper extracts are thought to reduce both glycemia in diabetic rats [43] and blood triglyceride levels [44]. Figure 5D shows the tight cluster correlation of GLs including MGs, DGs, TGs, MGDGs, DGDGs, SQDGs, and STs such as stigmasterol hexoside (SG: Hex) in six herbal teas. This figure shows that lemon peel has a higher amount of lipids, followed by ginger and fennel. The lipid molecular species DG (18:2/20:4) is slightly abundant in juniper, but other lipids showed lower concentrations. SQDG (16:0/18:0), SQDG (18:1/18:1), and DG (18:1/18:1) are abundant in fennel, but less abundant in rosehip. In addition, SQDG (16:0/18:1) was lower in rosehip, as shown in Figure 5D. Sterols are present in the lemon peel sample, which could be a factor in increasing the antioxidant capacity of milk after the addition of citrus peel to the ruminant diet [24]. Overall, the study highlights the beneficial effects of these herbal teas on human health and their lipidomic importance.

### 3.5. Identification and Characterization of Fatty Acid Esters of Hydroxy Fatty Acids (FAHFAs) and N-acyl-lysophosphatidylethanolamine (LPE-N) in Herbal Teas

**Figure 6:**
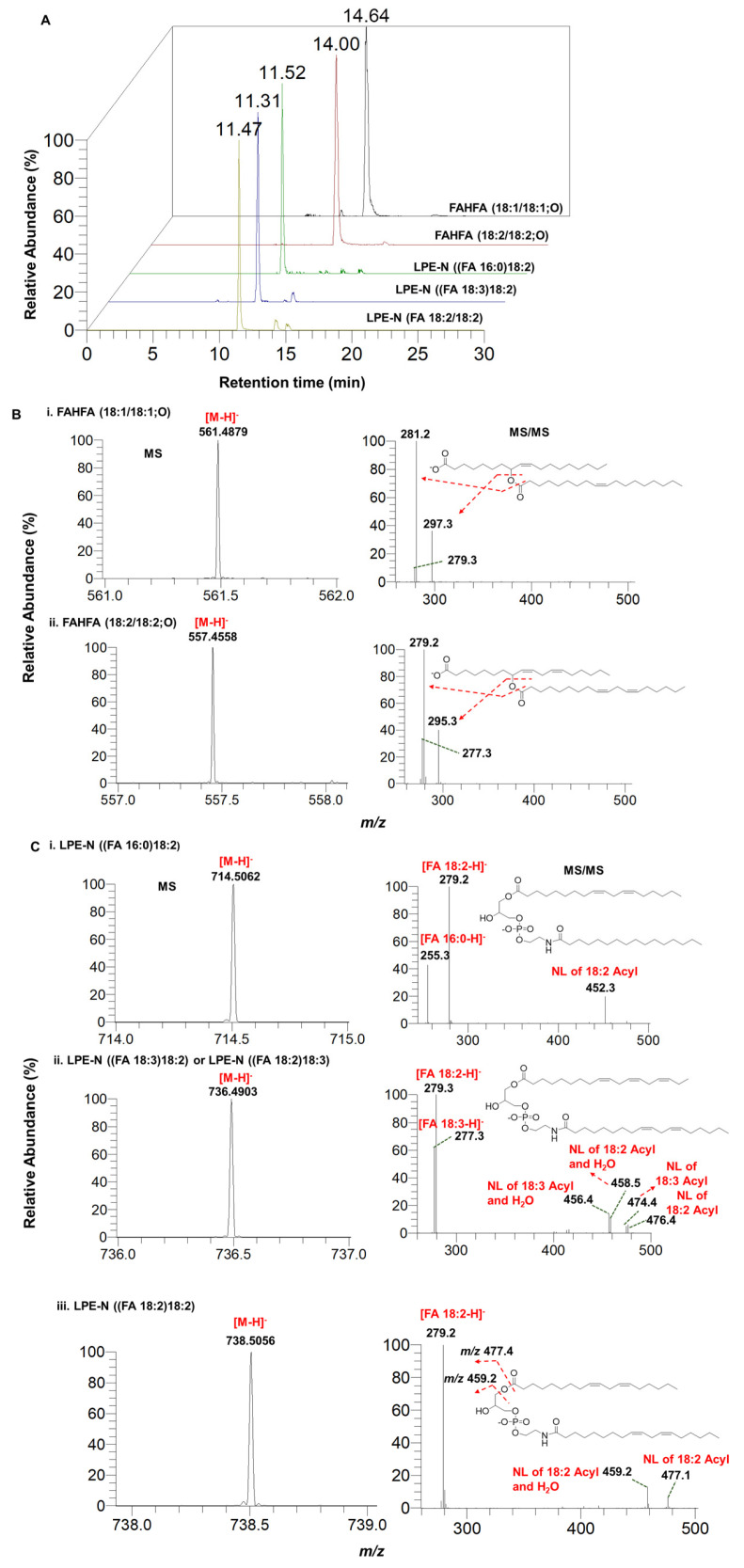
(**A**) Extracted ion chromatograms of fatty acid ester of hydroxy fatty acids (FAHFAs) and N-acyl-lysophosphatidylethanolamine (LPE-N), (**B**) MS and MS/MS spectra of FAHFAs (position of -OH is not determined in this study), and (**C**) MS and MS/MS spectra of LPE-Ns detected in herbal tea.

Hunting for the discovery of new metabolites in herbs using LC–MS, GC–MS, and NMR techniques has been widely investigated in the literature [45,46,47,48]. However, the lipid metabolite data in herbs are very limited. The comprehensive lipidomic analysis of six herbal teas resulted in the identification of two FAHFAs, namely FAHFA (18:1/18:1; O) and FAHFA (18:2/18:2; O), and three LPE-Ns, namely LPE-N ((FA 16:0)18:2), LPE-N ((FA 18:2) 18:2), and LPE-N ((FA 18:3) 18:2). The FAHFAs have been previously identified and characterized in various plant species, seeds, and fruits, including rice plants and nuts, and are considered to be new lipid classes with anti-diabetic and anti-inflammatory properties [49,50]. The identification of LPE-Ns has previously been reported in plant samples such as wheat [51]. However, to our knowledge, this is the first report on the descriptive identification and characterization of FAHFAs and LPE-N lipid species in these six herbal teas. The extracted ion chromatograms of the retention times of the identified lipid species are shown in Figure 6A. The high-resolution mass spectra (HRMS) and the MS/MS spectra of the two identified FAHFA lipids are shown in Figure 6B, and the three LPE-N lipid molecular species are shown in Figure 6C. FAHFAs and LPEN species were ionized to give [M–H]^−^ ions as their precursor ions. The molecule eluted at 14.64 min with an experimental *m*/*z* of 561.4879 ([C_36_H_65_O_4_–H]^−^, calculated *m*/*z*: 561.4887, mass error: −1.42 ppm) and the MS/MS spectra showed that [FA 18:1–H]^−^ was the major fragment ion (*m*/*z* 281.2), confirming that the detected compound was FAHFA (18:1/18:1; O), as shown in Figure 6B(i). The compound eluted at 14.00 min with an experimental *m*/*z* of 557.4558 ([C_36_H_61_O_4_–H]^−^, calculated *m*/*z*: 557.4575, mass error: −3.04 ppm), as shown in the HRMS and the MS/MS spectra, was [FA 18:2–H]^−^, the major fragment ion of *m*/*z* 279.2, suggesting that the detected compound was FAHFA (18:2/18:2; O), as shown in Figure 6B(ii). The lipid eluted at 11.52 min with an experimental *m*/*z* of 714.5062 ([C_39_H_73_NO_8_P–H]^−^, calculated *m*/*z*: 714.5078, mass error: −2.23 ppm), as shown in the HRMS and the MS/MS spectra, was [FA 18:2–H]^−^, the major fragment ion with an *m*/*z* of 279.2, and [FA 16:0–H]^−^ was also another fragmented ion at an *m*/*z* 255.3, suggesting that the detected compound was LPE-N ((FA 16:0) 18:2), as shown in Figure 6C(i). Similarly, the lipid metabolite eluted at 11.31 min with an experimental *m*/*z* of 736.4903 ([C_41_H_73_NO_8_P–H]^−^, calculated *m*/*z*: 736.4923, mass error: −2.71 ppm), as shown in HRMS and the MS/MS spectra, was [FA 18:2–H]^−^, the major fragment ion of *m*/*z* 279.3, and [FA 18:3–H]^−^ was another fragment ion at an *m*/*z* 277.3. The neutral loss of the 18:3 acyl and 18:2 acyl chains suggests that the detected molecule is isomeric in nature, such as LPE-N (FA 18:2) 18:3) or LPE-N ((FA 18:3) 18:2), as shown in Figure 6C(ii). Furthermore, the lipid molecule eluted at 11.47 min with an experimental *m*/*z* of 738.5056 ([C_41_H_71_NO_8_P–H]^−^, calculated *m*/*z*: 738.5078, mass error: −2.97 ppm), as shown in HRMS and the MS/MS spectra, was [FA 18:2–H]^−^, the major fragment ion at an *m*/*z* 279.2, indicating the detected lipid was LPE-N ((FA 18:2) 18:2), as shown in Figure 6C(iii). These fragmentation patterns are identical to our previous study for the identification and characterization of LPE-N molecular species [38]. 

The results of this analysis will provide valuable insights into the bioactive properties and industrial applications of these herbal teas in food production. Fennel, ginger, and juniper are commonly used as spices in the kitchen and have been shown to possess various biological effects [2,5,15]. Similarly, lemon peel, orange peel, and rosehip are used as foods and dietary supplements for both animals and humans [25,31]. These herbal teas have a wide range of pharmacological and industrial applications, some of which are mentioned in these studies [3,11,15,25,31]. Although this study has extensively investigated the lipidome of these herbal teas, it is important to acknowledge its limitations. The reported lipid concentrations are relative and depend on the amount of internal standard added, as well as the extraction procedure. In addition, factors such as origin, weather, and time of sampling may have affected the accuracy of the lipid compositions, which were not accounted for in this study. Therefore, only the six herbal teas mentioned in this study were considered for the comparison and correlation of lipid compositions. To provide a more comprehensive lipidomic analysis in the future, it will be necessary to compare the lipid compositions of different varieties of the same herbal teas separately.

## 4. Conclusions

The present study is believed to be the first to perform a comprehensive lipid composition analysis and provide a more accurate correlation of lipid molecular species for six herbal teas using a non-targeted LC–MS method. This investigation revealed several lipid classes, including approximately thirty distinct FFAs (SFAs, MUFAs, and PUFAs) containing both ω-3 and ω-6 fatty acids. In particular, juniper and fennel were found to be more important sources of different FFAs. Ginger is a rich source of SPs, lysophospholipids, and STs, while fennel is a rich source of ceramides and phospholipids, which have been shown to have positive effects on human health. Furthermore, this study has identified and characterized new lipid species in these herbal teas, such as FAHFA (18:1/18:1; O) and FAHFA (18:2/18:2; O), as well as several LPE-Ns. The application of the non-targeted LC–MS technique to explore the lipid sources in different samples such as food, plants, seeds, and animals is of great interest, as it can help to identify unexplored lipids. The identified lipid sources can be used to improve food varieties in the industry.

## Data Availability

The original contributions presented in the study are included in the article/Supplementary Materials, further inquiries can be directed to the corresponding authors.

## Supplementary Materials

The following supporting information can be downloaded at: https://www.mdpi.com/article/10.3390/foods13121877/s1, Table S1: Relative amount of lipid characterized in herbal tea.
